# Hysteresis Compensation and Sliding Mode Control with Perturbation Estimation for Piezoelectric Actuators

**DOI:** 10.3390/mi9050241

**Published:** 2018-05-16

**Authors:** Bingxiao Ding, Yangmin Li

**Affiliations:** 1 Department of Electromechanical Engineering, University of Macau, Taipa, Macao 999078, China; yb57419@umac.mo; 2 Department of Industrial and Systems Engineering, The Hong Kong Polytechnic University, Hung Hom, Hong Kong 999077, China; 3 Tianjin Key Laboratory for Advanced Mechatronic System Design and Intelligent Control, Tianjin University of Technology, Tianjin 300384, China

**Keywords:** micro-positioning, hysteresis compensation, sliding mode control, perturbation estimation

## Abstract

Based on the background of atomic force microscope (AFM) driven by piezoelectric actuators (PEAs), this paper proposes a sliding mode control coupled with an inverse Bouc–Wen (BW) hysteresis compensator to improve the positioning performance of PEAs. The intrinsic hysteresis and creep characteristics degrade the performance of the PEA and cause accuracy loss. Although creep effect can be eliminated by the closed-loop control approach, hysteresis effects need to be compensated and alleviated by hysteresis compensators. For the purpose of dealing with the estimation errors, unmodeled vibration, and disturbances, a sliding mode control with perturbation estimation (SMCPE) method is adopted to enhance the performance and robustness of the system. In order to validate the feasibility and performance of the proposed method, experimental studies are carried out, and the results show that the proposed controller performs better than a proportional-integral-derivative (PID) controller at 1 and 2 Hz, reducing error to 1.2% and 1.4%, respectively.

## 1. Introduction

**Figure 1:**
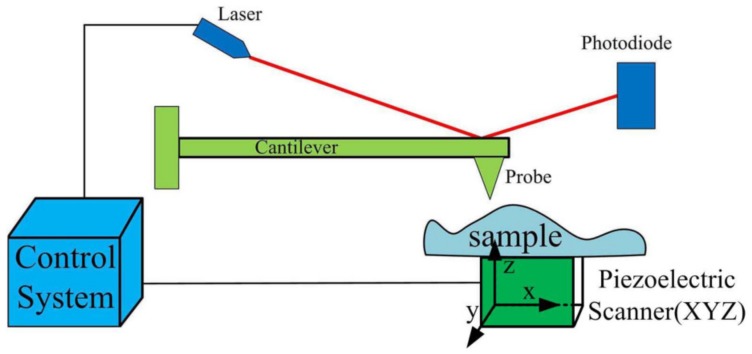
A schematic diagram of an atomic force microscope (AFM).

The atomic force microscope (AFM) is one of the most commonly used tools for imaging, measuring, and manipulating objects at the sub-micrometer level. It belongs to the class of scanning probe microscopy, which involves the utilization of a probe to scan sample surfaces, based on the principle of measuring the interactive force between the tip and the surface. Forces like Van dar Waals forces, electrostatic forces, and capillary forces are applied to the tip by the sample surface, resulting in the bending of the cantilever. Therefore, the surface topography of the sample can be acquired by recording the small deflections of the elastic cantilever [1,2]. As shown in Figure 1, the sample moves under the probe in the XYZ direction, and the probe moves up and down along the *Z*-direction. When the distance of the probe–surface changes, the cantilever will bend backwards or forwards, causing the position of the reflected point changes on the photodiode, and the surface topography is therefore recorded [3].

**Figure 2:**
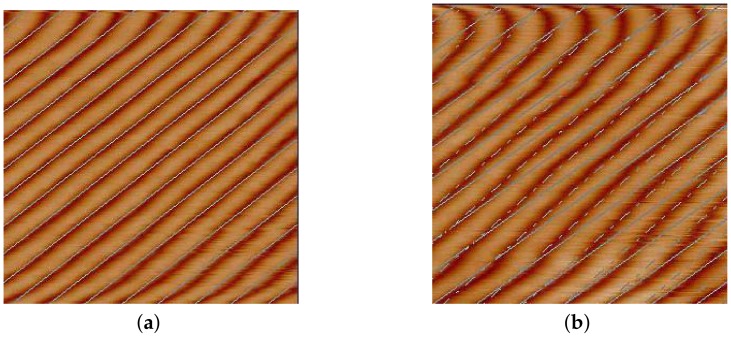
Measuring images with hysteresis and creep compensation vs. no compensation at a 0.5 Hz scanning rate. (**a**) With hysteresis and creep compensation. (**b**) Without hysteresis and creep compensation. Note: these two images are measured with a *CSPM5500* AFM.

Among piezoelectric actuators (PEAs), magnetostrictive actuators (MGAs), shape memory alloy (SMAs), and voice coil motors (VCMs), PEAs are the best choice as they can provide micro/nano resolution, fast response, and high output force in applications that require ultra-high precision motion [4,5]. However, the PEA exhibits nonlinearity characteristics, such as hysteresis and creep, and these phenomenons can decrease the positioning accuracy and affect the quality of measuring images. The negative effect caused by the nonlinearity characteristic can be validated by two images obtained with a *CSPM5500* AFM (Benyuan, Guangzhou, China), as depicted in Figure 2. Referring to it, we can conclude that the image quality of Figure 2a is better than that of Figure 2b, because Figure 2a is measured using hysteresis compensation and Figure 2b is obtained without nonlinearity compensation.

The compensation and alleviation of creep and hysteresis nonlinearity have thus become urgent for the purpose of obtaining higher image quality. Creep can be alleviated by a feedback controller, and hysteresis can be corrected by compensation techniques and a closed-loop control approach [6,7]. Currently, there are mainly three methods to alleviate hysteresis. Charge control can be adopted to correct the nonlinearity because of the existing linear relationship between dielectric displacement and PEA deformation. However, this method requires a specific charge drive circuit and may cause drift and saturation problems [8,9]. Closed-loop control with strain gauges, capacitive sensors, and laser sensors adopted to measure the output displacement of the PEA are utilized to improve the tracking precision. However, tracking performance is degraded with high input frequency signals. Some researchers have focused on modeling hysteresis nonlinearity based on the Duhem Model, the Bouc–Wen (BW) model, the Prandtl–Ishlinskii model, and the Preisach model [10,11,12,13] and have derived an inverse hysteresis model to compensate for nonlinearity using a feedforward control approach. Although the feedforward compensation control approach can reduce hysteresis to some degree, it cannot meet the requirements of high-precision applications.

In order to further improve the positioning accuracy of the PEA, this paper proposes a feedforward compensator based on an inverse hysteresis model combined with a feedback controller. The feedforward compensator is established based on a BW hysteresis model for the advantages of a few unknown parameters that can be easily identified using an intelligent algorithm. Sliding mode control with perturbation estimation is adopted to deal with parameter errors and external disturbances for the purpose of ensuring the positioning accuracy of the system. The rest of the paper is arranged as follows: Section 2 introduces the dynamic model based on the BW hysteresis model and identifies the parameters by utilizing a comprehensive learning particle swarm optimization (CLPSO) algorithm. The hysteresis compensator established based on the inverse BW hysteresis model is then proposed in Section 3. Lyapunov stability analysis of the proposed controller is presented in Section 4. Experimental studies are described in Section 5 to validate the performance of the controller. Section 6 summarizes this research.

## 2. Dynamic Modeling and Parameters Identification

### 2.1. Dynamic Modeling

The entire dynamic model of a PEA with BW hysteresis and perturbations can be established by using a spring-mass-damper model, as follows [14]:

(1) $$\begin{array}{c} m\ddot{x}+b\dot{x}+kx=k\left(d_{e}u-h\right)+P\\ \dot{h}=\alpha d_{e}\dot{u}-\beta \dot{u}\left|h|-\gamma \right|\dot{u}|h \end{array}$$

where *h* represents the hysteretic state variable, *u* is the input voltage, $m,b,d_{e}$, and *k* represent the equivalent mass, the damping coefficient, the piezoelectric coefficient, and stiffness, respectively; *P* denotes the external perturbations of the system caused by temperature, pre-load, and uncertainties of parameters. Besides that, the parameter α controls the hysteretic amplitude, while β and γ control the shapes of the hysteresis loop.

### 2.2. Parameter Identification

Methods adopted to identify the BW model parameters can be divided into two categories. One is based on the minimization of the error equation and includes the evolutionary algorithm (EA), differential evolution (DE), particle swarm optimization (PSO), and least square estimation (LSE); the other one is based on the nonlinear filtering method and includes the unscented Kalman filter [15].

In this paper, the CLPSO method is adopted to identify the parameters $m,b,d_{e},k,\alpha ,\beta$, and γ. CLPSO is one of variants of the PSO approach, which simulates the behaviour of a swarm, like bird or ant systems [16,17]. The fitness function also has an important effect on optimization results. There are mainly three types of fitness functions that can be adopted as an optimized objective function. The following one is selected in this paper for the benefits of having better performance and an identified response [18]:

(2) $$F\left(m,b,d_{e},k,\alpha ,\beta ,\gamma \right)=\frac{1}{N}\sum_{i=1}^{n}E_{i}^{2}$$

where

$$E_{i}=x_{i}-{x_{i}}^{bw}.$$

Here, *N* is the total number of sample points, and $E_{i}$ denotes the error between the *i*th experimental data $x_{i}$ and the *i*th simulation data $x_{i}^{bw}$ of the output displacement via establishing the BW model with identified parameters $m,b,d_{e},k,\alpha ,\beta ,\gamma$.

The identification procedures are listed as follows:

*Data Collection*: With the full-range-driven voltage applied to the PEA, the output displacement is measured and recorded. Hysteresis phenomenon is rate-dependent, namely the output of the system is sensitive to the variation of the input rate. This means that a higher frequency signal input results in a larger hysteresis loop generated [19]. Referring to the specification document of the adopted PEA, the amplitude of the input sinusoidal signal is set to 100 V with a 0.5 Hz frequency to stimulate the PEA.

*Parameters Identification*: After collecting the experimental data of the PEA, the CLPSO approach is adopted to search the model parameters which can closely match the simulation output to the experimental data through the established Simulink model.

**Figure 3:**
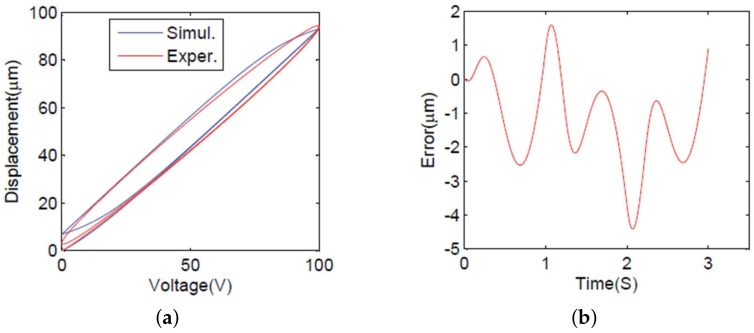
Hysteresis. (**a**) The experimental hysteresis loop and the simulation hysteresis loop. (**b**) Errors.

Based on obtained parameters shown in Table 1, the comparison between simulation hysteresis and experimental hysteresis is presented in Figure 3a, and the error between simulation results and experimental results are depicted in Figure 3b, which shows that the maximum deviation is about 4.2% with respect to the travel range of the PEA.

## 3. Hysteresis Compensation Based on the Inverse BW Model

As mentioned above, the intrinsic nonlinearity hysteresis characteristics can deteriorate the positioning performance of the PEA. Therefore, the hysteresis nonlinearity must be compensated and eliminated by the inverse hysteresis operator. The idea of compensating the hysteresis is to cascade the inverse feedforward hysteresis operator $H^{-1}$ with the actual hysteresis operator that is represented by *H* to obtain an identified mapping between the desired output $x_{d}\left(t\right)$ and the actual actuator response x(t). It can be described by the following equation:

(3) $$x\left(t\right)=H[H^{-1}\left[x_{d}\left(t\right)\right]]=x\left(t\right).$$

**Figure 4:**
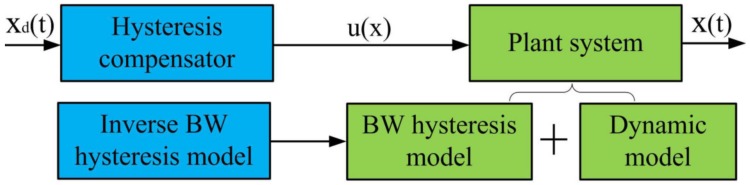
The principle to compensate hysteresis.

The principle to compensate the hysteresis is described in Figure 4. The hysteresis compensator is established based on the inverse BW hysteresis model and the plant system can be decomposed into the BW hysteresis model and the dynamic model of the PEA. After the desired input $x_{d}$ is applied to the hysteresis compensator, the obtained output voltage u(x) is adopted to drive the plant system and then output an experimental displacement x(t). Based on Equation (1), the u(x) can be described by the following equation:

(4) $$u\left(x\right)=\frac{m\ddot{x_{d}}+b\dot{x_{d}}+kx_{d}+kh+P}{kd_{e}}.$$

**Figure 5:**
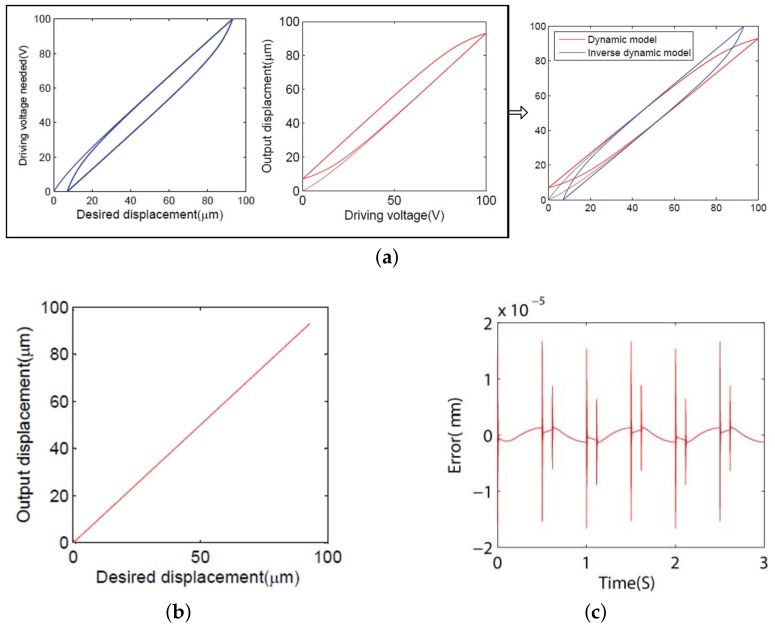
Simulation results with the inverse BW hysteresis compensator. (**a**) The hysteresis loop and inverse hysteresis loop.(**b**) The relationship between the desired displacement and the output displacement. (**c**) The error between the desired displacement and the output displacement after hysteresis compensation.

In order to validate the performance of the proposed compensator, the established model was implemented in MATLAB software (R2013b, MathWorks, Natick, MA, USA). The simulation results are presented in Figure 5, which illustrates the process of eliminating the hysteresis based on the inverse BW hysteresis compensator. Figure 5a shows that, after the desired displacement passes through the inverse BW model, the obtained output voltage signal is sent to the real system to cancel hysteresis problems. Figure 5b shows the relationship between the desired input displacement and the output displacement after hysteresis compensation, and the error is shown in Figure 5c. It can be learned that the PEA can be treated as a linear approximation system after hysteresis compensation.

## 4. The SMCPE Controller Coupled with Hysteresis Compensator

**Figure 6:**
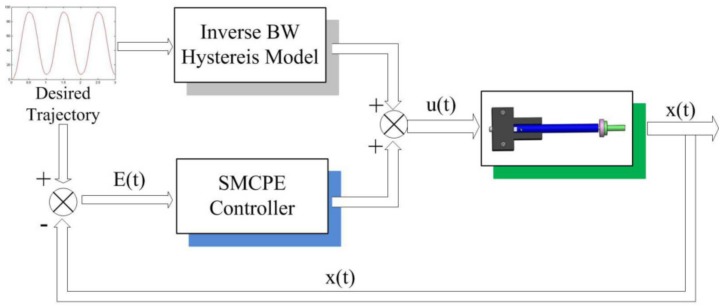
Schematic diagram of the tracking control system for piezoelectric actuators (PEAs).

This section mainly focuses on controller design and stability analysis. The principle of the proposed tracking control diagram is depicted in Figure 6. The hysteresis compensator can partially eliminate the nonlinearity hysteresis, and the system can be treated as a linear system approximately. To improve the robustness of the system for better tracking performance, the external/internal disturbance, system uncertainties, and other nonlinearities are viewed as a perturbation term (denoted by *P* in Equation (1)) and estimated by the perturbation technique. The perturbation estimation technique and sliding mode controller design process will be introduced in the following subsections.

### 4.1. The Perturbation Estimation Technique

This subsection briefly introduces the online perturbation estimation approach. The controllable canonical form of the nonlinear dynamic system can be described by the following equation [20]:

(5) $$x^{\left(n\right)}=f\left(X\right)+\Delta f\left(X\right)+\left[B\left(X\right)+\Delta B\left(X\right)\right]u\left(t\right)+d\left(t\right)$$

where $X_{i}$ ($X_{i}={\left[x_{i}^{\left(0\right)},x_{i}^{\left(1\right)},\cdots ,x_{i}^{\left(n_{i}-1\right)}\right]}^{T}\in R^{n_{i}}$, i=1,2,⋯,m, where $x^{\left(n\right)}=\left[x_{1}^{n_{1}},x_{2}^{n_{2}},\cdots ,x_{m}^{n_{m}}\right]\in R^{m}$ are *m* independent coordinates and *n* denotes the system order) is the state sub-vector, which forms the global state vector X = ${\left[X_{1}^{T},X_{2}^{T},\cdots ,X_{m}^{T}\right]}^{T}\in R^{r},r=\sum_{i=1}^{m}n_{i}$. The f = ${\left[f_{1},f_{2},\cdots ,f_{m}\right]}^{T}\in R^{m}$ and $\Delta f={\left[\Delta f_{1},\Delta f_{2},\cdots ,\Delta f_{m}\right]}^{T}\in R^{m}$ denote the nonlinear driving terms and their perturbations, respectively; $B=\left[b_{ij}\right]\in R^{m\times m}$ and $\Delta B=\left[\Delta b_{ij}\right]\in R^{m\times m}$, i,j=1,2,3,⋯,m represent the control gains and their uncertainties, respectively; the disturbance vector and control vector are described by the $d={\left[d_{1},d_{2},\cdots ,d_{m}\right]}^{T}\in R^{m}$ and $u={\left[u_{1},u_{2},\cdots ,u_{m}\right]}^{T}\in R^{m}$, respectively.

The uncertainty and parameter estimation errors of the system can be combined together to form the perturbation vector:

(6) $$\begin{array}{c} \Psi (X,t)=\Delta f+\Delta \mathrm{Bu}(t)+d(t)\\ =x^{\left(n\right)}-f-\mathrm{Bu}\left(t\right). \end{array}$$

The perturbation Ψ(X,t) can be estimated by the following equation:

(7) $$\Psi {\left(X,t\right)}_{estimated}=x_{calculated}^{\left(n\right)}-f-\mathrm{Bu}\left(t-T\right)$$

where *T* is the sampling interval, and u(t−T) denotes the control input in the previous time step. Theoretically, the $x_{calculated}^{\left(n\right)}$ can be computed by the Backward Euler method if the sampling time interval is very tiny.

(8) $$x^{n}=\frac{x^{\left(n-1\right)}\left(t\right)-x^{\left(n-1\right)}\left(t-T\right)}{T}.$$

In fact, u(t)≠u(t−T), so it can cause an approximate error using u(t−T) to replace u(t), and the measured displacement with noise can also result in errors. All these aforementioned errors can be viewed as unknown parameters about the system. To deal with these errors, the SMCPE is adopted as an enhanced version of the conventional SMC. The major contribution of SMCPE is to remove the requirement of estimation uncertainly bounds and replace it with a scheme for the online estimation of the perturbations [21,22].

### 4.2. System Description

Based on the above analysis, the state equation of this nonlinear system can be written as

(9) $$\dot{x}\left(t\right)=\mathrm{Ax}\left(t\right)+\mathrm{Bu}\left(t\right)+d\left(t\right).$$

Referring to Equation (1), the state variable $x\left(t\right)={\left[x_{1},x_{2}\right]}^{T}$ of the system is defined as below:

(10) $$\left\{\begin{array}{c} x_{1}=x\\ x_{2}=\dot{x_{1}} \end{array}\right.$$

with

(11) $$A=\left[\begin{array}{cc} 0 & 1\\ \theta_{1} & \theta_{2} \end{array}\right];B=\left[\begin{array}{c} 0\\ \theta_{3} \end{array}\right];d\left(t\right)=\left[\begin{array}{c} 0\\ P/m+\theta_{1}h \end{array}\right]$$

where $\theta_{1}=-k/m$, $\theta_{2}=-b/m$, $\theta_{3}=-kd_{e}/m$, and *h* denotes the hysteresis term. After compensation the hysteresis nonlinearity, the d(t) can be written as

(12) $$d\left(t\right)=\left[\begin{array}{c} 0\\ P/m+\theta_{1}\tilde{h} \end{array}\right]$$

where $\tilde{h}$ denotes the error between the actual hysteresis and the estimated hysteresis.

The auxiliary control input $u_{a}$ is defined to simplify the analysis.

(13) $$u=u_{a}+\frac{\hat{h}}{d_{e}}$$

where $\hat{h}=h-\tilde{h}$.

Thus, the system-dynamic Equation (1) can be derived as follows:

(14) $$\ddot{x}=\theta_{1}\;x+\theta_{2}\dot{x}+\theta_{3}\;u_{a}+\theta_{1}\tilde{h}+\frac{P}{m}.$$

The perturbation estimation function corresponding to the system can be defined as

(15) $$\Psi \left(x,t\right)=\theta_{1}\tilde{h}+\frac{P}{m}.$$

Referring to Equation (7), the estimated perturbation function $\Psi {\left(x,t\right)}_{est}$ can be written as

(16) $$\Psi {\left(x,t\right)}_{est}=\ddot{x}-\theta_{1}\;x-\theta_{2}\dot{x}-\theta_{3}\;u_{a}\left(t-T\right).$$

Defining $\tilde{\Psi }=\Psi \left(x,t\right)-\Psi {\left(x,t\right)}_{est}$, $\tilde{\Psi }$ is the error of actual perturbation function and its estimation. Therefore, Equation (14) can be written as follows:

(17) $$\ddot{x}=\theta_{1}\;x+\theta_{2}\dot{x}+\theta_{3}\;u_{a}+\tilde{\Psi }+\Psi {\left(x,t\right)}_{est}.$$

### 4.3. Sliding Mode Controller Design

The positioning tracking error of the system can be expressed as

(18) $$e=x_{1}-x_{d},$$

and the derivative of the tracking error is

(19) $$\dot{e}=x_{2}-\dot{x_{d}}.$$

Therefore, we define the sliding plane as a proportion-derivative type [23]:

(20) $$S=\lambda e+\dot{e}.$$

Therefore, the following equation can be derived:

(21) $$\dot{S}=\lambda \left(\dot{x}-\dot{x_{d}}\right)+\ddot{x}-\ddot{x_{d}}.$$

Additionally, the exponential reaching law is defined as follows [24]:

(22) $$\dot{S}=-kS-\eta sgn\left(S\right)+\tilde{\Psi }.$$

The control law can be derived as follows:

(23) $$u_{a}=\frac{1}{\theta_{3}}\left\{\ddot{x_{d}}-\theta_{1}x-\theta_{2}\dot{x}-\Psi {\left(x,t\right)}_{est}-kS-\eta sgn\left(S\right)-\lambda \dot{e}\right\}$$

where *k* is a positive constant parameter, and sgn(.) denotes the signum function. To guarantee the stability of the system and satisfy $\lim_{t\to \infty }e\left(t\right)=0$, the η must satisfy the following condition:

(24) $$\eta >|\tilde{\Psi }|.$$

### 4.4. Stability Analysis

The proof of the stability of the proposed control law is described in this subsection. Considering a positive definite Lyapunov function $V=\frac{1}{2}S^{2},$ the first derivative can be written as

(25) $$\begin{array}{c} \dot{V}=S\dot{S}\\ =S(-kS-\eta \mathrm{sgn}\left(S\right)+\tilde{\psi })\\ \leq -kS^{2}+\left(\left|\tilde{\psi }\right|-\eta \right)\left|S\right|\\ \leq 0 \end{array}.$$

Based on the above analysis, when t→∞, sliding surface S→0. Therefore, tracking error e→0 and asymptotical stability can be achieved. Therefore, the proposed control law can guarantee good tracking performance in theory. The discontinuity of the signum function may cause a chattering phenomenon in the control system. To eliminate this problem, the concept of a boundary layer is utilized to replace the signum function to smooth the control signal [25]. Therefore, the control law can be written as

(26) $$u=\frac{1}{\theta_{3}}\left\{\ddot{x_{d}}-\theta_{1}x-\theta_{2}\dot{x}-\Psi {\left(x,t\right)}_{est}-kS-\eta sat\left(\frac{S}{\varepsilon }\right)-\lambda \dot{e}\right\}+\frac{\hat{h}}{d_{e}}.$$

Here, ε is a positive constant that denotes the boundary layer thickness.

## 5. Performance Validation and Discussion

In this section, the experimental setup is introduced and experimental studies are described to validate the performance of the proposed controller.

### 5.1. Experimental Setup Configuration

**Figure 7:**
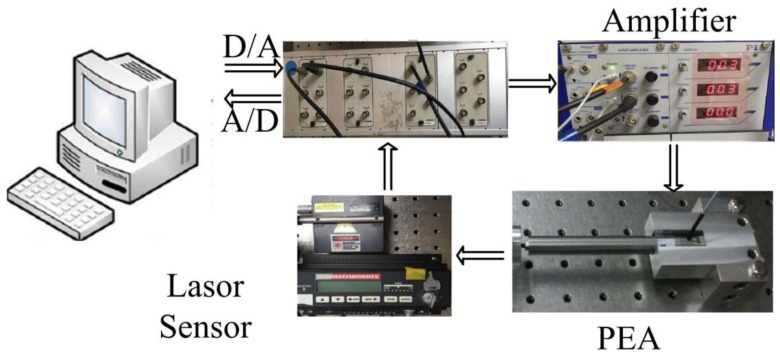
System block diagram.

A preloaded PEA *P-840.60* from Physik Instruments (PI, Karlsruhe, Germany) was adopted, with a nominal maximum travel range of 90 μm and an input voltage range of 0–100 V. The PEA was driven by a voltage amplifier with a gain ratio of 10. The displacement of the PEA was measured by the laser displacement sensor *Microtrak* II, *LTC-025-02*, from MTI Instrument, Inc., Albany, NY, USA. To suppress the external vibration, the PEA and laser sensor were mounted on an anti-vibration table. The system connection of the experimental setup is illustrated in Figure 7. The control block was designed and implemented via Simulink and the input signal through D/A block to drive the PEA. The measured analog signal was transferred to the Simulink through A/D block for recording and analyzing.

### 5.2. Experimental Results and Discussion

**Figure 8:**
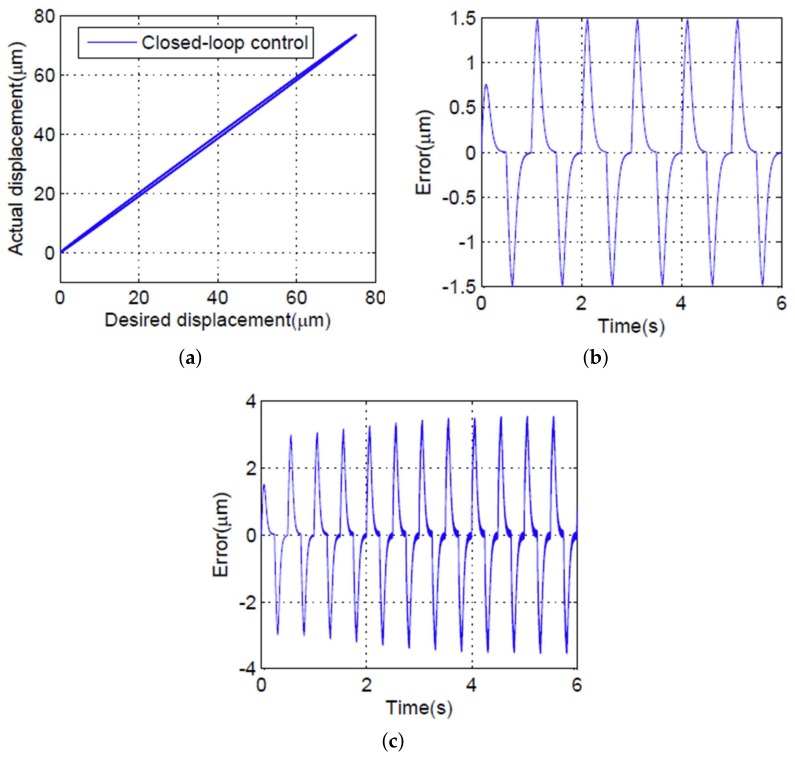
Experimental results with the feedforward hysteresis compensator and the PID controller. (**a**) The relationship between desired and actual displacement of the PEA under a 1 Hz sinusoidal input. (**b**) Tracking error in the case of a 1 Hz input signal. (**c**) Tracking error in the case of a 2 Hz input signal.

**Figure 9:**
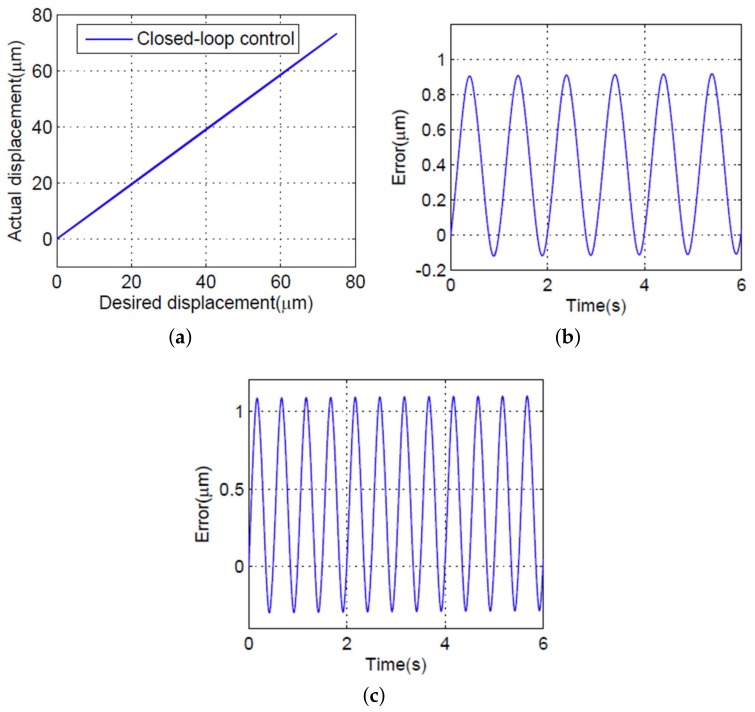
Experimental results with the feedforward hysteresis compensator and the proposed controller. (**a**) The relationship between the desired and the actual displacement of the PEA under a 1 Hz sinusoidal input. (**b**) Tracking error in the case of a 1 Hz input signal. (**c**) Tracking error in the case of a 2 Hz input signal.

To validate the performance of the proposed controller, experimental studies are described in this section. The proposed controller and the PID controller were compared. The value of the control parameters are listed in Table 2. During the experimental studies, sine wave signals with 0.5, 1, 1.5, and 2 Hz input signals were used to stimulate the PEA, and the maximum output displacement of PEA was 75 μm, some experimental results are shown in Figure 8 and Figure 9. Figure 8a shows the relationship between desired displacement and the actual output displacement with the PID controller under a 1 Hz input signal. The maximum absolute error was approximately 1.5 μm (2%) when the 1 Hz input frequency was applied, as shown in Figure 8b. As shown in Figure 8c, the error became larger and close to 3.9 μm (5.2%) when the 2 Hz input signal was applied. The relationship between desired displacement and the actual output displacement with the proposed controller is shown in Figure 9a, indicating an approximately linearity line under a 1 Hz input signal. The maximum absolute error was approximately 0.9 μm (1.2%) with a 1 Hz input signal, as shown in Figure 9b; the error was increased to 1.1 μm (1.4%) when the input frequency was up to 2 Hz.

We can conclude that the positioning performance of the proposed SMCPE approach is better than that of the PID controller, with an error decreasing to 1.2% and 1.4% at 1 and 2 Hz, respectively. Meanwhile, the positioning accuracy of the PEA performs better under a low input frequency compared with a high input frequency. For example, the tracking error of the PEA decreased from 5.2% to 2% with the PID controller and decreased from 1.4% to 1.2% with the proposed controller, with input frequency decreasing from 2 to 1 Hz, respectively, which means that the proposed controller cannot adapt to different input frequencies.

## 6. Conclusions

A SMCPE approach coupled with a hysteresis compensator based on the BW model is proposed in this paper for the purpose of improving the tracking performance of PEAs. The BW hysteresis model is utilized to model the hysteresis of the PEA for the benefit of a few unknown parameters, and the CLPSO approach is adopted to identify the parameters of the hysteresis model. Then, the inverse hysteresis model is derived to compensate for hysteresis nonlinearity. In order to improve the robustness of the system and obtain better tracking performance, the external disturbance, system uncertainties, and other nonlinearities are viewed as perturbation terms and estimated by the perturbation technique. The experimental results showed that the designed controller can provide superior tracking performance for PEAs with low input frequencies.

## Figures and Tables

**Table 1:** Identified parameters of the system with the Bouc–Wen (BW) hysteresis model.

Parameter	Value	Unit
*m*	0.015	Kg
*b*	0.01	N s/m
*k*	$1.1\times 10^{7}$	N/m
*d*	$1\times 10^{-6}$	m/V
α	0.4587	-
β	0.05	-
γ	0.0157	-

**Table 2:** Value of the control parameters.

PID	SMCPE
$k_{p}$ = 0.0002	λ = 1800
$k_{i}$ = 1.01 × $10^{-5}$	k = 2200
$k_{d}$ = 2.6 × $10^{-6}$	η = 1.2
∼	ϵ = 0.001
